# Perceived Implementation of Applied Behavior Analysis Techniques Among Teachers of Students with Autism Spectrum Disorder in Qatar

**DOI:** 10.3390/bs16061005

**Published:** 2026-06-16

**Authors:** Haifa Alhajri, Ali M. Alodat, Qais Al-Meqdad, Alanoud Binnoora

**Affiliations:** Department of Psychological Sciences, Qatar University, Doha P.O. Box 2713, Qatar; haalhajri@qu.edu.qa (H.A.); q.al-meqdad@qu.edu.qa (Q.A.-M.); a.binnoora@qu.edu.qa (A.B.)

**Keywords:** applied behavior analysis, autism spectrum disorder, teachers, implementation, Qatar, inclusive education

## Abstract

Applied Behavior Analysis (ABA) is recognized as one of the most evidence-based interventions for students with autism spectrum disorder (ASD), although its effectiveness relies on consistent classroom implementation by teachers. This study investigated the extent to which teachers of students with ASD in Qatar implement ABA techniques and whether implementation levels vary by gender, educational level, school type, years of experience, and teaching stage. A descriptive–analytical design was utilized on a sample of 155 teachers from government and private schools. Data were collected using the Applied Behavior Analysis Implementation Scale for Teachers of Students with ASD in Qatar (ABAIS-Qatar), a 26-item instrument developed and validated for this study across five dimensions. Teachers reported a high overall level of ABA implementation (M = 4.10, SD = 0.48). The Behavior Identification and Goal Setting and Strategy Application and Intervention dimensions received the highest ratings, while the Motivation and Corrective Procedures dimensions were rated at a moderate level. A five-way MANOVA revealed significant multivariate differences across years of experience and teaching stage. Post hoc analyses indicated that teachers with more than 15 years of experience reported significantly higher implementation of motivational and corrective procedures than those with 6–10 years of experience and that primary-stage teachers demonstrated superior classroom behavior management compared to intermediate-stage teachers. The findings have implications for teacher professional development and ABA training in both inclusive and specialist educational settings in Qatar.

## 1. Introduction

Autism spectrum disorder (ASD) is a neurodevelopmental condition characterized by persistent differences in social communication and interaction, as well as restricted and repetitive patterns of behavior and interests (American Psychiatric Association, 2022; UNICEF, 2025). ASD impacts various domains of functioning, including language development, emotional regulation, adaptive behavior, and participation in academic and social contexts. ASD is conceptualized as a spectrum, indicating substantial heterogeneity in presentation, severity, and support needs among individuals (Morsa et al., 2022; Russell et al., 2023). This variability has significant implications for educational practice, as learners with ASD may exhibit wide-ranging communication profiles, behavioral patterns, cognitive abilities, and responsiveness to intervention (Lord et al., 2020; Tomlinson, 2023).

In educational settings, these differences require individualized, flexible, and evidence-based instructional approaches that can be adapted to address diverse learner needs (Tomlinson, 2023). Students with ASD frequently benefit from structured teaching environments, explicit instruction, and systematic behavioral supports to facilitate learning and participation (Odom, 2019). In the absence of appropriate support, students with ASD often encounter challenges related to engagement, peer interaction, and academic achievement. Therefore, the effectiveness of educational provision depends not only on the availability of suitable interventions but also on their consistent, contextually relevant implementation in classroom settings (Bond et al., 2016; Watkins et al., 2019).

### 1.1. Applied Behavior Analysis in Autism Education

Applied Behavior Analysis (ABA) is among the most extensively researched and widely implemented intervention approaches for individuals with ASD (Honorato et al., 2024; Yu et al., 2020). Rooted in the principles of behaviorism, ABA investigates the relationship between environmental variables and behavior to increase socially significant behaviors and reduce those that hinder learning and functioning (Cooper et al., 2020). In educational contexts, ABA has become a well-established intervention for autism education due to its robust empirical foundation and adaptability to individual learners’ profiles (Gitimoghaddam et al., 2022). ABA-informed practices, including reinforcement procedures, task analysis, discrete-trial teaching, prompting, and other structured strategies, have proven their effectiveness in improving communication, adaptive behavior, social interaction, and academic engagement among students with ASD (Du et al., 2024).

The literature consistently supports the effectiveness of ABA-based interventions for children with ASD, especially when implementation is intensive, systematic, and tailored to individual needs. Empirical studies and reviews report positive effects on communication, emotional-social skills, adaptive behavior, and classroom functioning, although outcomes frequently depend on the quality, consistency, and dosage of intervention (Al Fandi & Zaharudin, 2023; Rodgers et al., 2020). Other researchers have demonstrated positive outcomes in language development, social skills, cognitive functioning, and adaptive behavior (Reichow et al., 2018; Rodgers et al., 2020). Others linked early behavioral interventions to improvements in cognitive and adaptive functioning among children with autism when implemented with adequate intensity and fidelity (Peters-Scheffer et al., 2011). However, the strength and consistency of this evidence are limited by methodological constraints, and findings require cautious interpretation (Vivanti et al., 2014).

Although there is substantial evidence base, ABA remains a subject of ongoing debate within the autism community and academic scholarship. Self-advocates for children with ASD and proponents of neurodiversity have raised ethical concerns about certain behaviorally intensive practices, particularly regarding their potential to prioritize compliance over the identity, well-being, and self-determination of individuals with ASD (Anderson, 2023; Kupferstein, 2018). Critics argue that some ABA procedures, especially those historically associated with aversive techniques, may conflict with a rights-based and neurodiversity-affirming approach to disability (Sandoval-Norton & Shkedy, 2019). In response, contemporary practitioners emphasize naturalistic, child-led, and exclusively positive approaches, focusing on quality of life and meaningful outcomes in addition to skill acquisition (Leaf et al., 2022; Schreibman et al., 2015). This study acknowledges these evolving perspectives and adopts a neutral, implementation-focused stance by examining the extent to which teachers report using ABA strategies, without making claims regarding their desirability or impact on individual students.

### 1.2. Teachers’ Role in ABA Implementation

Although ABA is often delivered in clinical settings by specialized practitioners, its implementation in school environments has gained increasing attention (Barnett et al., 2020). Classroom teachers are central to implementing behavioral supports in schools, as they interact with students daily and are responsible for translating intervention principles into practice during instruction and classroom routines (Al Fandi & Zaharudin, 2023; Jabri et al., 2025). Therefore, teachers’ understanding of ABA principles, confidence in employing behavior-analytic strategies, and access to appropriate supports are critical for successful implementation (Horner & Kittelman, 2021; Kos, 2021).

Effective implementation of ABA in classroom settings requires multiple competencies, including knowledge of behavioral principles, proficiency in implementing intervention strategies, and the ability to collect and interpret data to inform instruction (Horner & Sugai, 2015). Teachers must also adapt interventions to meet the needs of individual learners while maintaining consistency and fidelity. These requirements underscore the significance of both pre-service preparation and ongoing professional development in behavior-analytic practices (Lane et al., 2015; Sawyer et al., 2017).

Research indicates that teachers’ knowledge, beliefs, and self-efficacy substantially influence their use of evidence-based practices for students with ASD (Alhossein, 2021; Jennett et al., 2003). Teachers who possess confidence in their ability to implement behavioral interventions are more likely to apply them consistently and effectively. In contrast, limited knowledge or confidence may lead to inconsistent implementation or reliance on less targeted approaches (Chezan et al., 2023).

Teacher competence in ABA implementation is widely acknowledged as important; however, preparation in behavioral interventions for autism varies considerably across educational contexts (Jabri et al., 2025). Certain teacher education programs offer structured training in behavior analysis and autism-specific interventions, whereas others provide only minimal exposure to these methods (Petersson-Bloom et al., 2023).

Empirical studies indicate that inadequate training correlates with reduced implementation of individualized, data-driven interventions. Teachers with limited preparation often depend on general classroom management strategies instead of employing targeted behavioral interventions specifically designed for students with ASD (Barry et al., 2022). Conversely, teachers who receive specialized training are more likely to utilize evidence-based practices and report increased instructional confidence (Alhossein, 2021).

Professional development is essential for addressing gaps in teacher preparation. Effective training programs generally incorporate modeling, practice, feedback, and ongoing coaching to facilitate skill acquisition and retention (Taylor et al., 2005). Nevertheless, access to high-quality professional development remains inconsistent, especially within rapidly evolving educational systems (Shernoff et al., 2018).

### 1.3. Barriers to Implementation of ABA in Schools

ABA is supported by a robust evidence base; its implementation in classroom settings is frequently constrained by practical challenges. These barriers occur at multiple levels, including individual teacher factors, classroom conditions, and broader institutional structures (Alkaabi & Al-Maadid, 2025).

At the teacher level, insufficient training and limited confidence may impede effective implementation (Pickard et al., 2024). Large class sizes, diverse student needs, and time constraints often hinder the provision of individualized instruction and consistent reinforcement (Anderson, 2023). Moreover, resource limitations pose additional challenges, as teachers may lack access to specialized materials, data-collection tools, or support personnel, such as behavior analysts or special educators. Furthermore, the data-driven nature of ABA necessitates ongoing monitoring and analysis of student progress, which can be difficult to sustain alongside other instructional responsibilities (Cooper et al., 2020).

At the institutional level, the availability of support systems, leadership commitment, and school culture determines the extent to which ABA can be implemented effectively (Simpson, 2005). In addition, collaboration among teachers, specialists, and families is essential for maintaining consistency across settings and ensuring that interventions address student needs (Donaldson & Stahmer, 2014).

### 1.4. Theoretical Framework

The present study is grounded in the Active Implementation Framework (AIF), which was developed by Fixsen et al. (2005) through a comprehensive synthesis of the implementation of science literature. Implementation science examines the processes, factors, and conditions that influence the adoption, fidelity, and sustainability of evidence-based practices in real-world settings (Fixsen et al., 2005). In educational contexts, AIF has been widely utilized to investigate how evidence-based interventions, including ABA-based practices, transition from controlled research environments to routine classroom application (Odom, 2019).

According to the AIF, successful implementation of evidence-based practices in educational settings depends not only on the quality of the intervention, but also on three interacting categories of implementation drivers: competency drivers, organizational drivers, and leadership drivers (Fixsen et al., 2005). Competency drivers involve the selection, training, and ongoing coaching of practitioners, specifically teachers in this context, who are responsible for delivering the intervention. Organizational drivers pertain to administrative structures, resource allocation, and systemic support that facilitate or hinder implementation. Leadership drivers refer to the adaptive and technical decision-making capacities necessary to manage change within complex institutional environments. These drivers function interactively; weaknesses in one domain may be offset by strengths in another, but sustained implementation fidelity requires alignment across all three domains.

The AIF further delineates four distinct stages of implementation: exploration, installation, initial implementation, and full implementation. This framework emphasizes that implementation is a process that unfolds over time rather than a single event (Fixsen et al., 2005). The temporal aspect is particularly pertinent to this study, as years of professional experience have emerged as a significant predictor of variation in ABA implementation levels.

By utilizing this framework, the study conceptualizes ABA implementation in Qatari schools as a systemic process influenced by teachers’ professional preparation, institutional support, and contextual factors, rather than solely as an individual-level competency. The aim of this study aligns directly with the competency driver domain of the AIF, addressing the specific knowledge and behavioral repertoire required for teachers to implement ABA with fidelity. Demographic variables such as years of experience, educational level, and school type serve as proxies for the competency and organizational drivers identified by the framework, enabling analysis of how these implementation conditions differ across the Qatari educational context.

However, because these AIF drivers were not directly measured in the present study, interpretations based on them remain inferential rather than empirically verified. Subsequent research should include direct measurement of coaching access, organizational support, and leadership engagement to fully operationalize the framework.

### 1.5. Study Context

ASD constitutes a significant developmental disability in Qatar, with national epidemiological data estimating a prevalence of 1.14% among children aged 6 to 11 years (Alshaban et al., 2019). The referenced large-scale study identified 1393 children with existing ASD diagnoses and confirmed further cases through systematic screening and diagnostic evaluation. These results suggest that ASD prevalence in Qatar is considerably higher than previously reported local estimates, a trend corroborated by initial reports of the study’s findings (Furfaro, 2019).

In response, Qatar’s education system has increasingly prioritized inclusive schooling and the provision of appropriate support for students with disabilities (Darwish et al., 2025; Jabri et al., 2025). National policy frameworks, such as Qatar National Vision 2030, emphasize quality education, human development, and the inclusion of learners with multiple needs in mainstream educational settings (Alodat et al., 2025; Al-Zyoud et al., 2025).

Despite these policy commitments, recent research indicates that teachers in inclusive classrooms in Qatar continue to face challenges in effectively addressing their students’ diverse academic and behavioral needs (Jabri et al., 2025). Qatar’s educational system consists of government schools serving Qatari nationals and adhering to the national curriculum, alongside a large and diverse private school sector that primarily serves expatriate students. Both sectors have increasingly integrated students with ASD; however, systemic differences in resources, staff preparation, and institutional support may result in variations in implementation practices. Additionally, ABA training is not consistently incorporated into teacher education programs in Qatar, leading many practitioners to acquire relevant skills through in-service professional development rather than pre-service training. This context positions Qatar as a distinctive and significant setting for examining ABA implementation and indicates that findings may not be directly generalizable to other national or cultural contexts. On the other hand, although the broader literature supports the use of ABA, there is limited empirical evidence regarding teachers’ knowledge of ABA and the extent to which those strategies are implemented in Qatari classrooms. Specifically, little is known about how implementation varies according to teachers’ professional preparation and experience.

### 1.6. The Current Study

This study examines the extent to which teachers of students with ASD in Qatar implement ABA techniques in classroom practice. It also investigates whether the level of implementation varies by selected teacher and contextual characteristics, such as gender, educational attainment, school type, years of experience, and teaching stage. The study is guided by the following research questions:

RQ1: To what extent do teachers of students with autism spectrum disorder in Qatar apply applied behavior analysis techniques?

RQ2: Are there significant differences in the extent to which teachers of students with autism spectrum disorder in Qatar apply applied behavior analysis techniques, by gender, educational level, type of school, years of experience, and teaching stage?

## 2. Materials and Methods

### 2.1. Research Design

This study used a descriptive–analytical research design, a method well-suited for capturing and measuring real-world trends (Loeb et al., 2017). By systematically gathering and analyzing quantitative data, this approach offers an accurate depiction of current practices and reveals how various contextual factors may shape them.

### 2.2. Participants

A total of 155 teachers working with students with ASD in Qatari schools participated in the study during the 2024–2025 academic year. The mean age of participants was 34.6 years (SD = 7.8). The majority were female (*n* = 123, 79.4%), while male teachers comprised 20.6% of the sample (*n* = 32). Most teachers held a bachelor’s degree (*n* = 101, 65.2%), followed by postgraduate qualifications (*n* = 49, 31.6%), and a small proportion held a diploma (*n* = 5, 3.2%). More than half of the participants were employed in specialist schools (*n* = 90, 58.1%), while the remainder worked in inclusive settings (*n* = 65, 41.9%). The largest proportion of teachers had 1–5 years of teaching experience (*n* = 54, 34.8%), followed by 6–10 years (*n* = 42, 27.1%), more than 15 years (*n* = 32, 20.6%), and 11–15 years (*n* = 27, 17.4%). Most participants worked at the primary level (*n* = 102, 65.8%), with fewer at the secondary (*n* = 31, 20.0%) and intermediate (*n* = 22, 14.2%) stages.

### 2.3. Measures

Several instruments have been developed to assess teacher competencies or self-efficacy related to autism education, such as the Autism Self-Efficacy Scale for Teachers (Ruble et al., 2013) and the Abilities in Behavior Assessment and Intervention for Teachers-Revised (Dutt et al., 2019). However, none of these tools were designed to measure the extent to which classroom teachers implement specific ABA techniques in naturalistic school settings. Additionally, no validated Arabic-language instrument exists to assess ABA implementation among teachers in the Gulf region or in broader Arab educational contexts (Jabri et al., 2025). Existing English-language instruments are not directly transferable to the Qatari context due to differences in educational structures, cultural norms, instructional practices, and the linguistic needs of a professional population for whom Arabic is the primary language; therefore, the development of a context-specific instrument was warranted.

The Applied Behavior Analysis Implementation Scale for Teachers of Students with ASD in Qatar (ABAIS-Qatar) was developed specifically for this study to facilitate data collection. The five dimensions of the scale were derived from the foundational principles of ABA articulated by Baer et al. (1968) and further elaborated by Cooper et al. (2020). The ABAIS-Qatar consists of 26 items distributed across five dimensions: (1) Behavior Identification and Goal Setting (6 items), (2) Strategy Application and Intervention (6 items), (3) Reinforcement and Punishment (6 items), (4) Motivation and Corrective Procedures (4 items), and (5) Classroom Behavior Management (4 items). Responses were recorded on a five-point Likert-type scale ranging from 1 (never implemented) to 5 (largely implemented), with higher scores indicating greater ABA implementation. All items were positively worded.

Mean scores were interpreted using a classification scheme derived by dividing the scale range (4 points) by the number of response categories (5), resulting in an interval width of 0.80. The classification boundaries were as follows: very low (1.00–1.80), low (1.81–2.60), moderate (2.61–3.40), high (3.41–4.20), and very high (4.21–5.00).

Content validity was established through systematic expert review. A panel of nine academics with expertise in educational psychology and psychological counseling evaluated the initial version of the scale for content representativeness, construct relevance, and linguistic clarity. Items were retained if at least 80% of panel members agreed to include them. After adjudication, minor linguistic revisions were incorporated based on reviewer feedback, and the scale was finalized at 26 items. Construct validity was assessed through item analysis using data from a pilot sample of 25 teachers drawn from the study population but excluded from the main sample. Pearson correlation coefficients were calculated between each item and its respective dimension score, as well as between each item and the total scale score. Item-dimension correlations ranged from 0.628 to 0.879, and item-total correlations ranged from 0.404 to 0.698, with all values statistically significant at either *p* < 0.05 or *p* < 0.01. All coefficients exceeded the minimum acceptable threshold of 0.30 (Kline, 2000), indicating that each item contributed meaningfully to its designated dimension and to the scale as a whole. Exploratory factor analysis (EFA) was not performed on the current sample due to the limited pilot sample size (*n* = 25), which is insufficient for stable factor solutions (Tabachnick & Fidell, 2019). Subsequent studies should utilize both EFA and confirmatory factor analysis (CFA) with a larger, independent sample to rigorously assess the hypothesized five-factor structure of the ABAIS-Qatar.

Scale reliability was evaluated using two complementary approaches applied to the pilot sample data. Internal consistency was estimated using Cronbach’s alpha, which yielded a total-scale coefficient of 0.873. At the dimension level, alpha values ranged from 0.715 (Reinforcement and Punishment) to 0.858 (Strategy Application and Intervention), with the remaining dimensions producing coefficients of 0.851 (Behavior Identification and Goal Setting), 0.758 (Motivation and Corrective Procedures), and 0.796 (Classroom Behavior Management). Split-half reliability was also assessed, yielding a coefficient of 0.916 for the total scale, with dimension-level values ranging from 0.771 (Reinforcement and Punishment) to 0.872 (Classroom Behavior Management). All coefficients exceeded the widely accepted reliability threshold of 0.70 (Hogan, 2019), indicating that the ABAIS-Qatar demonstrates sufficient internal consistency and temporal stability for quantitative research.

After confirming the instrument’s psychometric adequacy, the finalized ABAIS-Qatar was converted into an electronic format using Google Forms. The survey included an introductory statement describing the study’s purpose, assurances of participant anonymity, and explicit instructions for responding to each item. To facilitate self-administration, each of the five dimensions was clearly labeled and briefly explained within the survey, enabling teachers to complete the instrument independently without prior training or the researchers’ assistance.

### 2.4. Data Collection

After obtaining ethical approval (QU-IRB 127/2025-EM) from Qatar University, schools enrolling students with autism spectrum disorder (ASD) were identified using the Ministry of Education database. Institutional emails were sent to 105 schools, inviting them to participate by distributing the survey link to teachers working with students with ASD. Follow-up emails and telephone calls were made to encourage participation. Of the schools contacted, 70 responded, resulting in a school-level response rate of 66.7%.

Participant recruitment used a nonprobability volunteer sampling approach, in which teachers self-selected to participate after receiving the survey link at their schools. This method is suitable when the research objective does not require inferential generalizations associated with probability-based sampling, and when accessibility and willingness to participate are primary considerations in sample formation (Murairwa, 2015). In accordance with this design, participation was entirely voluntary, responses were submitted anonymously, and no incentives were provided. A potential limitation of this approach is self-selection bias. Teachers who voluntarily participated may have greater familiarity with or interest in ABA, which could result in higher reported implementation levels compared to the broader population of teachers working with students with ASD in Qatar. This limitation should be taken into account when interpreting the results.

### 2.5. Statistical Analysis

Statistical analyses were performed using SPSS (version 28). For the first research question, descriptive statistics, including means and standard deviations, were calculated for each dimension of the ABA implementation scale and for the total scale score. For the second research question, a five-way Multivariate Analysis of Variance (MANOVA) was used to examine differences across all five scale dimensions based on gender, educational level, school type, years of experience, and teaching stage. Prior to conducting the MANOVA, key statistical assumptions were evaluated. Multivariate normality was assessed using Mardia’s test, and results indicated no significant departure from normality. Homogeneity of covariance matrices was examined using Box’s M test (M = 148.32, F = 1.21, *p* = 0.074), which yielded a non-significant result, indicating that the assumption was not violated. Multicollinearity among the five dependent variables was assessed through correlational analysis; intercorrelations were all below 0.70, indicating acceptable independence among dimensions. Subsequently, a five-way univariate Analysis of Variance (ANOVA) was applied to the total scale score to evaluate the overall effect of these variables. When significant multivariate or univariate effects were found, Scheffé post hoc tests were used to determine the direction and significance of pairwise mean differences. Effect sizes are reported as partial eta-squared (η^2^p) for all significant ANOVA results.

## 3. Results

### 3.1. Extent of ABA Implementation

To identify the extent to which teachers of students with ASD in Qatar apply ABA techniques, means and standard deviations were computed for each of the five scale dimensions and for the total scale. Results are presented in Table 1.

Overall, teachers reported a high level of ABA implementation (M = 4.10, SD = 0.48). At the dimension level, means ranged from 3.07 to 4.40. Behavior Identification and Goal Setting ranked highest (M = 4.40, SD = 0.65), followed by Strategy Application and Intervention (M = 4.36, SD = 0.67). Reinforcement and Punishment and Classroom Behavior Management were jointly ranked third, each with a mean of 4.18 (SDs = 0.66 and 0.76, respectively). Motivation and Corrective Procedures had the lowest mean across dimensions (M = 3.07, SD = 1.02), but still within the moderate range.

Regarding the Behavior Identification and Goal Setting dimension, item means ranged from 4.06 to 4.60 (see Appendix A, Table A1). The highest rating was observed for precision in identifying children’s behavior (M = 4.60, SD = 0.74), followed by attention to student–environment interaction when setting behavioral goals (M = 4.55, SD = 0.72). The lowest-rated item in this dimension was perceived ease and flexibility in identifying target behaviors (M = 4.06, SD = 1.02), which remained within the high implementation range.

For the Strategy Application and Intervention dimension, item means ranged from 4.09 to 4.58 (see Appendix A, Table A2). Prioritizing behavioral problems when planning interventions received the highest rating (M = 4.58, SD = 0.75), followed by modeling skills within chaining strategies (M = 4.54, SD = 0.80). Perceived freedom to implement ABA strategies without institutional constraints yielded the lowest rating within this dimension (M = 4.09, SD = 1.08), though it remained within the high range.

In the Reinforcement and Punishment dimension, item means ranged from 3.92 to 4.61 (see Appendix A, Table A3). Reinforcing the desired behavior was incompatible with the undesired behavior, which received the highest rating (M = 4.61, SD = 0.73), indicating that teachers frequently used differential reinforcement. Withholding reinforcement following undesired behavior received the lowest rating within this dimension (M = 3.92, SD = 1.11) yet also remained within the high implementation range.

The Motivation and Corrective Procedures dimension yielded the lowest item-level scores overall, with all four items falling within the moderate implementation means ranging from 2.82 to 3.32 (see Appendix A, Table A4). Prompting students with advance notice of reinforcement received the highest item mean within this dimension (M = 3.32, SD = 1.40), followed by verbal reprimand for repeated undesirable behavior (M = 3.24, SD = 1.30). Temporary removal from preferred peers received the lowest mean (M = 2.82, SD = 1.32). The relatively large standard deviations across these items indicate considerable individual variability in teachers’ use of motivational and corrective procedures.

In the Classroom Behavior Management dimension, item means ranged from 3.94 to 4.34 (see Appendix A, Table A5). Student involvement in selecting reinforcers received the highest rating (M = 4.34, SD = 1.01), followed by the organization of environmental factors relevant to behavior modification programs (M = 4.29, SD = 0.83). Response cost, defined as removing the student from the reinforcing environment, yielded the lowest mean item score within this dimension (M = 3.94, SD = 1.04) but remained within the high-implementation range.

### 3.2. Differences by Demographic Variables

To explore whether implementation levels differ based on gender, educational level, school type, years of experience, and teaching stage, descriptive statistics were first calculated for each subgroup across all five scale dimensions. A Five-Way Multivariate Analysis of Variance (MANOVA) was subsequently conducted to examine group differences across all five ABA dimensions simultaneously. Results are presented in Table 2.

The Five-Way MANOVA revealed statistically significant multivariate effects for years of experience (Wilks’ Λ = 0.151, F(5, 143) = 4.307, *p* = 0.001) and teaching stage (Wilks’ Λ = 0.085, F(5, 142) = 2.428, *p* = 0.038). No significant multivariate effects were observed for gender (Hotelling’s Trace = 0.014, F(5, 141) = 0.384, *p* = 0.859), educational level (Wilks’ Λ = 0.978, F(10, 282) = 0.313, *p* = 0.978), or school type (Hotelling’s Trace = 0.009, F(5, 141) = 0.255, *p* = 0.937). To identify the specific dimensions driving the significant multivariate effects, a Five-Way univariate Analysis of Variance (ANOVA) was conducted for each dimension separately. Results are presented in Table 3.

The ANOVA results at the dimension level showed that years of experience significantly affected the Motivation and Corrective Procedures dimension (F(3, 145) = 4.051, *p* = 0.008, η^2^p = 0.077, indicating a medium effect). Teaching stage was significant for Classroom Behavior Management (F(2, 145) = 4.735, *p* = 0.010, η^2^p = 0.061, indicating a small-to-medium effect). No other combinations reached statistical significance. Table 4 presents effect sizes for the two significant effects.

To determine the direction of the significant effect of years of experience on Motivation and Corrective Procedures, Scheffé post hoc comparisons were conducted (see Appendix B, Table A7). The analysis identified a statistically significant pairwise difference between teachers with 6–10 years of experience (M = 2.810) and those with more than 15 years of experience (M = 3.578; mean difference = −0.768, *p* < 0.05), with more experienced teachers reporting significantly higher implementation of motivational and corrective procedures. No other pairwise differences were statistically significant.

Scheffé post hoc comparisons were similarly conducted to examine the significant effect of teaching stage on Classroom Behavior Management (see Appendix B, Table A8). Primary-stage teachers (M = 4.267) reported significantly higher implementation than intermediate-stage teachers (M = 3.784; mean difference = 0.483, *p* < 0.05). Differences between primary and secondary stages (mean difference = 0.114, *p* > 0.05) and between intermediate and secondary stages (mean difference = −0.369, *p* > 0.05) did not reach statistical significance. To complement the multivariate analyses, a Five-Way ANOVA was conducted on the total ABA implementation scale score as a function of all five demographic variables. Results are presented in Table 5.

Regarding total scale means by subgroup, female teachers (M = 4.038, SD = 0.494) and male teachers (M = 4.032, SD = 0.397) reported nearly identical levels of implementation. For educational level subgroups, means ranged from 3.947 (Diploma) to 4.054 (Bachelor’s Degree). Teachers in inclusive and specialist schools reported similar total scale means of 4.035 (SD = 0.434) and 4.040 (SD = 0.529), respectively. Across experience groups, means ranged from 4.002 (1–5 years) to 4.128 (more than 15 years), and across teaching stages from 3.953 (secondary) to 4.074 (primary). The Five-Way ANOVA showed no statistically significant differences in total ABA implementation based on gender (F(1, 145) = 0.698, *p* = 0.405), education level (F(2, 145) = 0.680, *p* = 0.508), school type (F(1, 145) = 0.023, *p* = 0.879), experience years (F(3, 145) = 0.872, *p* = 0.457), or teaching stage (F(2, 145) = 1.768, *p* = 0.174). Although some significant differences appeared at the dimension level for experience and teaching stage, overall ABA implementation did not differ significantly across the demographic variables examined.

## 4. Discussion

This study explored teachers’ perceptions of how they implement ABA techniques with students with ASD in Qatar. It also examined whether this perceived implementation varies across demographic and contextual factors. Teachers reported a generally high level of ABA use, aligning with the study’s descriptive goals. However, since the data are self-reported rather than collected through direct observation or fidelity checks, the findings primarily reflect teachers’ views of their classroom practices rather than verified implementation. Specifically, the uniformly high scores across most dimensions suggest the potential presence of social desirability bias, in which respondents may report higher levels of implementation than they actually practice to present themselves favorably (Podsakoff et al., 2003). Additionally, common-method variance may be a concern because both predictor variables and outcomes were collected using the same self-report instrument (Harman, 1976). Future research should employ multi-method designs, such as direct classroom observation, to validate these self-reported patterns. Previous research consistently shows a gap between reported and actual use of evidence-based practices, highlighting the need for caution in interpreting self-report data (Horner & Kittelman, 2021; Chezan et al., 2023).

Using the AIF (Fixsen et al., 2005), the results suggest an early or developing phase of implementation. Teachers seem to have embraced essential ABA concepts and regularly use foundational strategies. However, without direct measures of practitioner skills, organizational infrastructure, or leadership support, it is difficult to assess implementation accuracy, fidelity, or sustainability (Odom, 2019). Therefore, the findings primarily reflect perceived adoption and involvement, rather than full or high-fidelity implementation.

Analysis across the five ABAIS Qatar dimensions shows significant variation that the high overall score hides. Teachers indicated very strong implementation in Behavior Identification, Goal Setting, Strategy Application, and Intervention. This suggests that ABA practices that are procedural, observable, and easily integrated into routine instruction are more effectively enacted (Cooper et al., 2020; Lane et al., 2015). In the AIF, these domains represent foundational skills that are more readily gained through traditional training and professional experience.

In contrast, Motivation and Corrective Procedures were rated as moderate, exhibiting significant variability among teachers. This finding represents a key outcome of the study and merits further analysis. Several factors may explain these comparatively lower ratings. From a training standpoint, motivating operations, shaping procedures, and mild corrective techniques are among the most technically demanding aspects of ABA, typically necessitating supervised practice and behavioral skills training to achieve proficiency (Cooper et al., 2020). Organizationally, the implementation of consequence-based strategies, such as response cost and contingent removal, may be restricted by school-level policies that limit the use of reductive procedures or require additional documentation and approval. Culturally, the application of corrective or punitive procedures may be viewed as inconsistent with relational norms in Qatari educational settings, where teacher-student relationships are generally guided by values of respect and positive regard. Furthermore, some of these procedures, including verbal reprimand and temporary peer removal, have been critically evaluated from neurodiversity and rights-based perspectives (Anderson, 2023), potentially contributing to practitioner ambivalence. These factors indicate that enhancing implementation in this domain requires policy clarification, cultural alignment, and ongoing professional reflection.

Demographic analyses reinforce this understanding. Consistent with the MANOVA results, there were no significant differences in overall perceived implementation across gender, educational level, or school type. This likely reflects shared exposure to professional norms or training rather than equal implementation skills (Alhossein, 2021; Barry et al., 2022). Conversely, teachers with over 15 years of experience reported notably higher implementation of motivation and corrective procedures. This indicates that accumulated experience enhances adaptive expertise through better situational judgment and flexible decision-making in complex classroom situations (Kos, 2021). Additionally, higher levels of classroom behavior management at the primary level suggest that organizational and instructional contexts affect implementation feasibility, particularly through features such as more structured routines, closer teacher–student interactions, and greater flexibility in behavior support practices (Horner & Sugai, 2015).

Overall, the results show that ABA practices are broadly acknowledged and documented in Qatari schools, but their application varies across different areas. In the AIF, this pattern suggests a system moving from initial adoption to more comprehensive implementation, highlighting the importance of enhancing competency, organizational capacity, and leadership to maintain consistent, high-quality practice.

## 5. Limitations

The findings of this study should be interpreted within defined limitations. First, the research assessed teachers’ perceived use of ABA practices rather than conducting direct classroom observations; therefore, the results pertain to self-reported implementation engagement rather than procedural fidelity. The consistently high scores across most dimensions suggest that social desirability bias and common method variance may have inflated reported implementation levels (Podsakoff et al., 2003). Future studies should employ observational measures and behavioral skills assessments to corroborate self-reported data. Second, the cross-sectional design provides only a single time-point snapshot of perceived implementation. Third, the use of nonprobability volunteer sampling restricts statistical generalizability. Self-selected participants may constitute a subset of teachers with greater familiarity with ABA, which could explain the elevated implementation scores observed. Fourth, although the ABAIS-Qatar demonstrated acceptable initial psychometric properties, neither exploratory nor confirmatory factor analysis was conducted due to the limited pilot sample size. Such analyses are necessary to formally validate the instrument’s five-factor structure. Finally, key AIF drivers, including coaching intensity, organizational infrastructure, and leadership engagement, were not directly measured. As a result, theoretical interpretations involving these constructs remain inferential rather than empirically substantiated.

## 6. Practical Implications

The findings suggest that enhancing ABA implementation in Qatar requires shifting from general adoption to more comprehensive, higher-quality practices. Professional development initiatives should prioritize advanced competencies, particularly in motivation and corrective procedures, and should incorporate ongoing coaching, modeling, and performance feedback (Sawyer et al., 2017; Taylor et al., 2005). Organizational structures must facilitate consistent application by providing protected planning time, effective data-collection tools, and opportunities for collaboration with specialists (Donaldson & Stahmer, 2014). Active leadership engagement is essential for coordinating resources and fostering a culture of continuous improvement (Fixsen et al., 2005; Shernoff et al., 2018). The lower implementation scores among intermediate-stage teachers in Classroom Behavior Management highlight the necessity for targeted support at this stage, which may be best addressed through differentiated professional development rather than uniform approaches. Additionally, in light of ongoing ethical debates regarding certain ABA procedures, professional development should incorporate critical reflection on practice, uphold the rights and dignity of students with ASD, and simultaneously advance technical proficiency.

## 7. Conclusions

This study advances understanding of ABA implementation by examining teachers’ perceptions of techniques employed in educational settings for students with ASD in Qatar. Although teachers generally reported high levels of ABA application, implementation varied across domains, with foundational practices reported more consistently than advanced procedures. The AIF suggests that implementation is in an early or transitional phase. The findings indicate that domain-level analysis provides a more sensitive assessment of implementation maturity than aggregate scores. These results underscore that the effectiveness of evidence-based practices depends on the alignment of practitioner competency, organizational support, and leadership. As a preliminary investigation, these findings should be interpreted as descriptive and exploratory. Future research should incorporate observational measures, larger and more representative samples, and formal validation of the ABAIS-Qatar instrument through factor analysis to provide a more comprehensive understanding of ABA implementation quality in Qatari and broader Gulf educational contexts.

## Figures and Tables

**Table 1:** Descriptive Statistics for ABA Implementation Dimensions and Total Scale.

Rank	No.	Dimension	M	SD	Level
1	1	Behavior Identification & Goal Setting	4.40	0.65	Very High
2	2	Strategy Application & Intervention	4.36	0.67	Very High
3	3	Reinforcement & Punishment	4.18	0.66	High
3	5	Classroom Behavior Management	4.18	0.76	High
5	4	Motivation & Corrective Procedures	3.07	1.02	Moderate
		Total Scale	4.10	0.48	High

Note. M = mean; SD = standard deviation. Classification: Very High (4.21–5.00); High (3.41–4.20); Moderate (2.61–3.40).

**Table 2:** Five-Way MANOVA Results for ABA Implementation Dimensions by Demographic Variables.

Variable	Test Statistic	Value	F	Hypothesis df	Error df	*p*
Gender	Hotelling’s Trace	0.014	0.384	5	141	0.859
Educational Level	Wilks’ Lambda	0.978	0.313	10	282	0.978
School Type	Hotelling’s Trace	0.009	0.255	5	141	0.937
Years of Experience	Wilks’ Lambda	0.151	4.307	5	143	0.001 *
Teaching Stage	Wilks’ Lambda	0.085	2.428	5	142	0.038 *

Note. * *p* < 0.05.

**Table 3:** Five-Way ANOVA Results for ABA Implementation by Dimension and Demographic Variable.

Source	Dimension	SS	df	MS	F	*p*
Gender	Behavior Identification & Goal Setting	0.528	1	0.528	1.212	0.273
	Strategy Application & Intervention	0.343	1	0.343	0.728	0.395
	Reinforcement & Punishment	0.112	1	0.112	0.253	0.616
	Motivation & Corrective Procedures	0.269	1	0.269	0.274	0.601
	Classroom Behavior Management	0.765	1	0.765	1.338	0.249
Educational Level	Behavior Identification & Goal Setting	0.641	2	0.320	0.735	0.481
	Strategy Application & Intervention	0.375	2	0.187	0.397	0.673
	Reinforcement & Punishment	0.373	2	0.187	0.423	0.656
	Motivation & Corrective Procedures	0.098	2	0.049	0.050	0.951
	Classroom Behavior Management	1.195	2	0.598	1.046	0.354
School Type	Behavior Identification & Goal Setting	0.045	1	0.045	0.102	0.750
	Strategy Application & Intervention	0.008	1	0.008	0.018	0.894
	Reinforcement & Punishment	0.008	1	0.008	0.018	0.893
	Motivation & Corrective Procedures	1.095	1	1.095	1.116	0.293
	Classroom Behavior Management	0.084	1	0.084	0.146	0.703
Years of Experience	Behavior Identification & Goal Setting	0.530	3	0.177	0.405	0.749
	Strategy Application & Intervention	0.790	3	0.263	0.558	0.643
	Reinforcement & Punishment	1.728	3	0.576	1.304	0.275
	Motivation & Corrective Procedures	11.918	3	3.973	4.051	0.008 *
	Classroom Behavior Management	0.981	3	0.327	0.572	0.634
Teaching Stage	Behavior Identification & Goal Setting	0.284	2	0.142	0.326	0.722
	Strategy Application & Intervention	0.315	2	0.158	0.334	0.716
	Reinforcement & Punishment	0.366	2	0.183	0.414	0.662
	Motivation & Corrective Procedures	3.188	2	1.594	1.625	0.200
	Classroom Behavior Management	5.411	2	2.706	4.735	0.010 *
Error	Behavior Identification & Goal Setting	63.173	145	0.436		
	Strategy Application & Intervention	68.371	145	0.472		
	Reinforcement & Punishment	64.021	145	0.442		
	Motivation & Corrective Procedures	142.213	145	0.981		
	Classroom Behavior Management	82.856	145	0.571		

Note. SS = sum of squares; df = degrees of freedom; MS = mean square. * *p* < 0.05.

**Table 4:** Effect Sizes for Significant ANOVA Results.

Source	Dimension	SS	df	F	*p*	η^2^p
Years of Experience	Motivation & Corrective Procedures	11.918	3	4.051	0.008 *	0.077
Teaching Stage	Classroom Behavior Management	5.411	2	4.735	0.010 *	0.061

Note. η^2^p = partial eta-squared. * *p* < 0.05. Effect size benchmarks: small = 0.01, medium = 0.06, large = 0.14 (Cohen, 1988).

**Table 5:** Five-Way ANOVA Results for Total ABA Implementation Scale Score by Demographic Variables.

Source	SS	df	MS	F	*p*
Gender	0.160	1	0.160	0.698	0.405
Educational Level	0.312	2	0.156	0.680	0.508
School Type	0.005	1	0.005	0.023	0.879
Years of Experience	0.601	3	0.200	0.872	0.457
Teaching Stage	0.812	2	0.406	1.768	0.174
Error	33.311	145	0.230		
Corrected Total	34.718	154			

Note. SS = sum of squares; df = degrees of freedom; MS = mean square. No variable reached statistical significance at α = 0.05.

## Data Availability

Data are available upon reasonable request from the corresponding author.

## Appendix A

Table A1, Table A2, Table A3, Table A4 and Table A5 present the item-level means, standard deviations, and implementation level ratings for each of the five dimensions of the ABAIS-Qatar, supplementing the dimension-level results reported in the main text.

**Table A1:** Item-Level Descriptive Statistics for the Behavior Identification and Goal Setting Dimension.

Rank	No.	Item	M	SD	Level
1	1	I am precise in identifying the child’s behavior, whether positive or negative.	4.60	0.74	VH
2	2	I pay considerable attention to the student’s interaction with the environment when setting behavioral goals.	4.55	0.72	VH
3	5	I continually evaluate the effectiveness of the behavior modification strategy I employ.	4.43	0.83	VH
4	4	I assess the target behavior across multiple settings to confirm it warrants modification.	4.41	0.91	VH
5	3	I define the target behavior precisely, measuring no more than one behavior at a time.	4.35	0.89	VH
6	6	I feel at ease and flexible when identifying the target behavior.	4.06	1.02	H
		Total	4.40	0.65	VH

Note. VH = Very High; H = High.

**Table A2:** Item-Level Descriptive Statistics for the Strategy Application and Intervention Dimension.

Rank	No.	Item	M	SD	Level
1	9	I prioritize the student’s behavioral problems when intervening, beginning with the most critical.	4.58	0.75	VH
2	11	I model skills that the student cannot perform within a chaining strategy.	4.54	0.80	VH
3	7	I focus on addressing observable and measurable behavior.	4.43	0.84	VH
4	8	I implement extinction by gradually reducing behavior through the withdrawal of reinforcers.	4.29	0.89	VH
5	10	I fade prompts gradually to prevent complete cessation of the target response.	4.23	0.88	VH
6	12	I have full autonomy to implement ABA strategies in school without restriction.	4.09	1.08	H
		Total	4.36	0.67	VH

Note. VH = Very High; H = High.

**Table A3:** Item-Level Descriptive Statistics for the Reinforcement and Punishment Dimension.

Rank	No.	Item	M	SD	Level
1	16	I reinforce the student for performing the desired behavior that is the opposite of the undesired behavior.	4.61	0.73	VH
2	13	I frequently use edible reinforcers because of their strong effect on target behaviors.	4.18	0.96	H
2	17	I frequently use tangible reinforcers (e.g., providing a toy/sticker after task completion).	4.18	0.98	H
4	15	I reduce undesirable behavior by modifying the environmental conditions in which it occurs.	4.12	0.99	H
5	18	I prefer using novel reinforcers.	4.06	0.95	H
6	14	I withhold reinforcement when the student engages in undesirable behavior.	3.92	1.11	H
		Total	4.18	0.66	H

Note. VH = Very High; H = High.

**Table A4:** Item-Level Descriptive Statistics for the Motivation and Corrective Procedures Dimension.

Rank	No.	Item	M	SD	Level
1	19	I prompt the student to perform the desired behavior with a cue that reinforcement will follow.	3.32	1.40	M
2	21	I verbally reprimand the student when undesirable behavior occurs repeatedly.	3.24	1.30	M
3	20	I reinforce the student’s performance during behavior shaping 3–5 times before advancing to the next level.	2.90	1.33	M
4	22	I temporarily remove the student from preferred peers in the classroom when undesirable behavior occurs.	2.82	1.32	M
		Total	3.07	1.02	M

Note. M = Moderate.

**Table A5:** Item-Level Descriptive Statistics for the Classroom Behavior Management Dimension.

Rank	No.	Item	M	SD	Level
1	23	I involve the student in selecting reinforcers for performing the desired behavior.	4.34	1.01	VH
2	25	I organize environmental factors that influence behavior modification programs.	4.29	0.83	VH
3	26	I easily redirect the student immediately after undesirable behavior, reminding them of acceptable conduct and requiring them to repair any harm.	4.14	0.97	H
4	24	I stop undesirable behavior by removing the student from the reinforcing environment.	3.94	1.04	H
		Total	4.18	0.76	H

Note. VH = Very High; H = High.

## Appendix B

Table A6, Table A7 and Table A8 present supplementary statistical data supporting the analyses reported in the Results section. Table A6 provides subgroup means and standard deviations for all five dimensions. Table A7 and Table A8 present the Scheffé post hoc comparison matrices for years of experience and teaching stage, respectively.

**Table A6:** Descriptive Statistics for ABA Implementation Dimensions by Demographic Variables.

Variable	Category	M1	SD	M2	SD	M3	SD	M4	SD	M5	SD
Gender	Female	4.382	0.687	4.347	0.690	4.192	0.684	3.104	1.003	4.165	0.808
	Male	4.474	0.472	4.411	0.609	4.120	0.560	2.938	1.081	4.219	0.564
Educational Level	Bachelor’s	4.431	0.605	4.373	0.646	4.210	0.626	3.052	1.051	4.203	0.733
	Diploma	4.333	1.061	4.133	0.923	3.967	0.711	3.300	1.304	4.000	0.984
	Postgraduate	4.347	0.697	4.357	0.712	4.133	0.726	3.082	0.935	4.138	0.812
School Type	Inclusive	4.394	0.621	4.350	0.647	4.143	0.611	3.139	0.980	4.147	0.747
	Specialist	4.410	0.688	4.374	0.713	4.226	0.723	2.973	1.069	4.215	0.788
Years of Experience	1–5 years	4.358	0.683	4.327	0.699	4.207	0.689	2.986	0.971	4.134	0.769
	6–10 years	4.385	0.619	4.361	0.593	4.333	0.532	2.810	1.000	4.173	0.807
	11–15 years	4.395	0.557	4.494	0.507	4.074	0.621	3.037	0.987	4.176	0.906
	>15 years	4.500	0.713	4.302	0.844	4.010	0.761	3.578	1.013	4.250	0.564
Teaching Stage	Primary	4.405	0.708	4.364	0.719	4.222	0.720	3.110	1.021	4.267	0.755
	Intermediate	4.417	0.556	4.356	0.573	4.144	0.571	3.216	0.940	3.784	0.974
	Secondary	4.376	0.500	4.349	0.597	4.054	0.488	2.831	1.056	4.153	0.511

Note. M1 = Behavior Identification & Goal Setting; M2 = Strategy Application & Intervention; M3 = Reinforcement & Punishment; M4 = Motivation & Corrective Procedures; M5 = Classroom Behavior Management. SD = standard deviation.

**Table A7:** Scheffé Post Hoc Comparisons for Dimension 4 (Motivation & Corrective Procedures) by Years of Experience.

Years of Experience	M	1–5 Years	6–10 Years	11–15 Years	>15 Years
1–5 years	2.986		0.176	−0.051	−0.592
6–10 years	2.810			−0.227	−0.768 *
11–15 years	3.037				−0.541
>15 years	3.578				

Note. Values represent mean differences. * *p* < 0.05.

**Table A8:** Scheffé Post Hoc Comparisons for Dimension 5 (Classroom Behavior Management) by Teaching Stage.

Teaching Stage	M	Primary	Intermediate	Secondary
Primary	4.267		0.483 *	0.114
Intermediate	3.784			−0.369
Secondary	4.153			

Note. Values represent mean differences. * *p* < 0.05.
